# Injectable silicone rubber for ocular implantation after evisceration

**DOI:** 10.1371/journal.pone.0193448

**Published:** 2018-03-23

**Authors:** Peng Fei Zheng, Qi Sheng You, Qian Li, Hong Yan Deng, Ian Y. H. Wong, Xiao Yan Peng

**Affiliations:** 1 Department of Ophthalmology, Beijing Tongren Eye Center, Beijing Tongren Hospital, Capital Medical University, Beijing, China; 2 Beijing Institute of Ophthalmology, Beijing Tongren Eye Center, Beijing Tongren Hospital, Capital Medical University, Beijing Ophthalmology and Visual Science Key Lab, Beijing, China; 3 Beijing Stomatology Hospital, Capital Medical University, Beijing, China; 4 Department of Ophthalmology, University of Hong Kong, Hong Kong, Hong Kong; Bascom Palmer Eye Institute, UNITED STATES

## Abstract

**Objective:**

To investigate the usefulness of addition type liquid silicone rubber (ATLSR) as injectable implant after evisceration to maintain the eyeball volume in an animal experiment.

**Methods:**

Twelve adult New Zealand white rabbits were included. One eye of each rabbit was randomly selected for evisceration with the fellow eye as control. ATLSR was injected to fill the eyeball socket after evisceration. In vivo observation and photographs were performed up to 24 weeks post-op. Two rabbits were sacrificed respectively at post-operative week 1, 2, 4, 8, 12 and 24. After enucleation, the vertical, horizontal and sagittal diameters of the experimental eyeballs were measured and compared with the control eyes. Histopathological studies were performed to evaluate signs of inflammation.

**Results:**

Cornea remained clear throughout the observation period despite mild epithelial edema and neovascularization. Compared to the control eyes, the experimental eyes were significantly smaller in vertical diameter (17.00±1.17 vs. 17.54±1.11 mm, P<0.001), but larger in sagittal diameter (16.85±1.48 vs. 16.40±1.38 mm, P = 0.008), and had no significant difference in horizontal diameter (17.49±1.53 vs. 17.64±1.21 mm, P = 0.34). Postoperative inflammation was observed at one week after surgery, which peaked at 2–3 weeks, then regressed gradually. At week 12 and week 24, most of the inflammatory cells disappeared with some residual plasma cells and eosinophils.

**Conclusion:**

Injectable addition type silicon rubber may be a good choice for ocular implantation after evisceration, maintaining eyeball volume and cosmetically satisfactory when compared to the fellow eye. Spontaneous regression of inflammation implied good biocompatibility for at least 24 weeks.

## 1.0 Introduction

Removal of painful blind eyes, cosmetically unacceptable blind eyes, and medically uncontrolled endophthalmitis as a result of severe trauma or disease can be accomplished by either evisceration or enucleation [1,2]. Evisceration is faster, less complex, and associated with less disruption of the surrounding orbital tissues, better maintenance of extraocular muscles motility, leading to superior cosmetic and functional results [3,4]. An ocular implant is usually needed to restore the orbit volume after evisceration or enucleation. Commonly used ocular implants are solid spheres of different materials such as porous polyethylene or coralline hydroxyapatite [5–8]. Reported complications include implant exposure, conjunctival thinning, discharge, implant infection and post-enucleation/evisceration socket syndrome (PESS) [9–12]. Several surgical techniques have been described to achieve better appearance and prosthesis motility as well as less complications [13–15]. When possible, an ocular implant should preferably be placed in the cornea-scleral shell after the removal of the ocular contents (in-situ placement), which in turn results in improved appearance and prosthesis motility [16]. Evisceration with corneal sparing was achieved through a relaxing sclerotomy incision and the implant would then be placed in the evisceration scleral shell [16]. However, implanting a solid sphere of appropriate size is technically challenging: the scleral cavity must be opened widely to accommodate the sphere during surgery, and it would be difficult for the implant to fit in without an adequate coverage by the donor sclera. Liquefied implants injected through small incisions may be an alternative when trying to avoid the mentioned problems [17]. Silicone rubber, a well-tolerated biomaterial [18,19], is commonly used in surgical implants. Addition type liquid silicone rubber (ATLSR) is composed of liquefied monomer and catalyzer. When mixed with the catalyzer, the monomer cross-links, solidifies, rending it easy to shape as well as physicochemically stable [20–22]. It has grown into a commonly used substance for making external prosthesis. In the current study, we investigate the applicability of ATLSR as an injectable implant after evisceration to maintain the eyeball volume in an animal experiment.

## 2.0 Materials and methods

### 2.1 Addition type liquid silicone rubber

ATLSR (Silagum-light) was obtained from DMG Chemisch-Pharmazeutische Fabrik GmbH. The ingredients include addition curing vinyl polysiloxanes, hydrogen polysiloxanes, fillers, pigments, additives, and platinum catalyst. The ingredients are pre-filled in an auto-mix cartridge. When injected, the materials are mixed in a mixing tip attached to the cartridge. Then addition polymerization starts within 2 minutes after mixing up and solidifies within 3 minutes and 30 seconds.

### 2.2 Animal experiments

The Animal Ethical Committee of the Capital Medical University reviewed and approved the animal experiments (No. of approval AEEI-2014-133, S1 and S2 Files)) and the study was carried out according to the Association for Research in Vision and Ophthalmology (ARVO) Statement for the Use of Animals in Ophthalmic and Vision Research.

Twelve adult New Zealand white rabbits were included in the study. Each rabbit was assigned a unique number (in consecutive order) for identification. For rabbits with an assigned odd number as identification number, either the right or left eye was randomly selected as the study eye, and the fellow eye as control eye. For even numbered rabbits, we selected the opposite laterality as study and control eye according to the previous odd-numbered rabbit. The animal experiments were carried out in the animal laboratory of the Capital Medical University. An experienced retina surgeon who was also certified and experienced in animal experiments performed all the surgical procedures. A professional animal anesthesiologist in the lab carried out general anesthesia, which was induced and maintained by intramuscular injection of 30mg/kg ketamine. Most of the surgical procedures were completed within 20 minutes. In three out of 12 rabbits, the surgical procedure took more than 20 minutes. For these three rabbits, a second injection of 15mg/kg ketamine was performed to extend the anesthesia effects. Povidone–iodine solution was used for disinfection after anesthesia. A metal speculum was used to open the eyelids for surgical procedure. A superior conjunctiva peritomy was performed from 10 to 2 o’clock. A sclerotomy parallel to the limbus was performed at 2mm posterior to the limbus, extending about 8mm length. An evisceration spoon was inserted into the suprachoroidal space to separate the choroid and sclera, and all the ocular contents were removed including the vitreous, choroid, retina, lens and iris. Any remaining choroidal tissue remnants and hemorrhage were removed with cotton swabs. The incision was partially closed with a 7-0-polygalactin suture, leaving 3mm gap for injection of ATLSR. The ATLSR was injected to fill the eyeball completely until overflowing was noted. It takes 2–3 minutes for the silicone rubber to solidify. The overflowing silicone rubber was removed with scissors. The sclera and conjunctiva incision were then closed with 7–0 polygalactin sutures. Tobramycin and dexamethasone ophthalmic ointment was applied to the eye post-operatively once daily for five days.

### 2.3 Post-operative clinical evaluation and histological study

The animals were observed daily for two weeks post-operatively to monitor for postoperative pain or infection. The signs of pain included hiding in a corner of the cage and reluctance to move, rough grinding of teeth, heavily breathing, or loss of appetite. The anterior segment photographs of the operated eyes were taken once every three days. Two rabbits were sacrificed at each time point at post-operative week 1, 2, 4, 8, 12 and 24 respectively. The rabbits were euthanized by intravenous injection of sodium pentobarbital (100mg/Kg). Both eyes were enucleated within 5 minutes after death. Both the operated experimental eyes and the fellow control eyes were measured for vertical, horizontal and sagittal diameters using calipers. The experimental eyes were fixed with 10% formalin for 72 hours. A central cross section was submitted for paraffin embedding. The sections were stained with hematoxylin and eosin and were examined by an ophthalmic pathologist under light microscope (Olympus, X51, Tokyo, Japan). Any abnormalities including signs of inflammation were recorded using a digitized image system (Moticam 2006, 2.0M pixel USB2.0, Motic China Group, Co. Ltd., Xiamen, China).

### 2.4 Statistical analysis

Statistical analysis was performed using SPSS for Windows (version 22.0; IBM-SPSS, Chicago, IL, USA). The paired Student t test was used to compare the diameters of the experimental eyes and the control eyes. P-values were based on 2-sided tests, with values less than 0.05 considered statistically significant.

## 3.0 Results

### 3.1 Postoperative clinical evaluation

**Fig 1:**
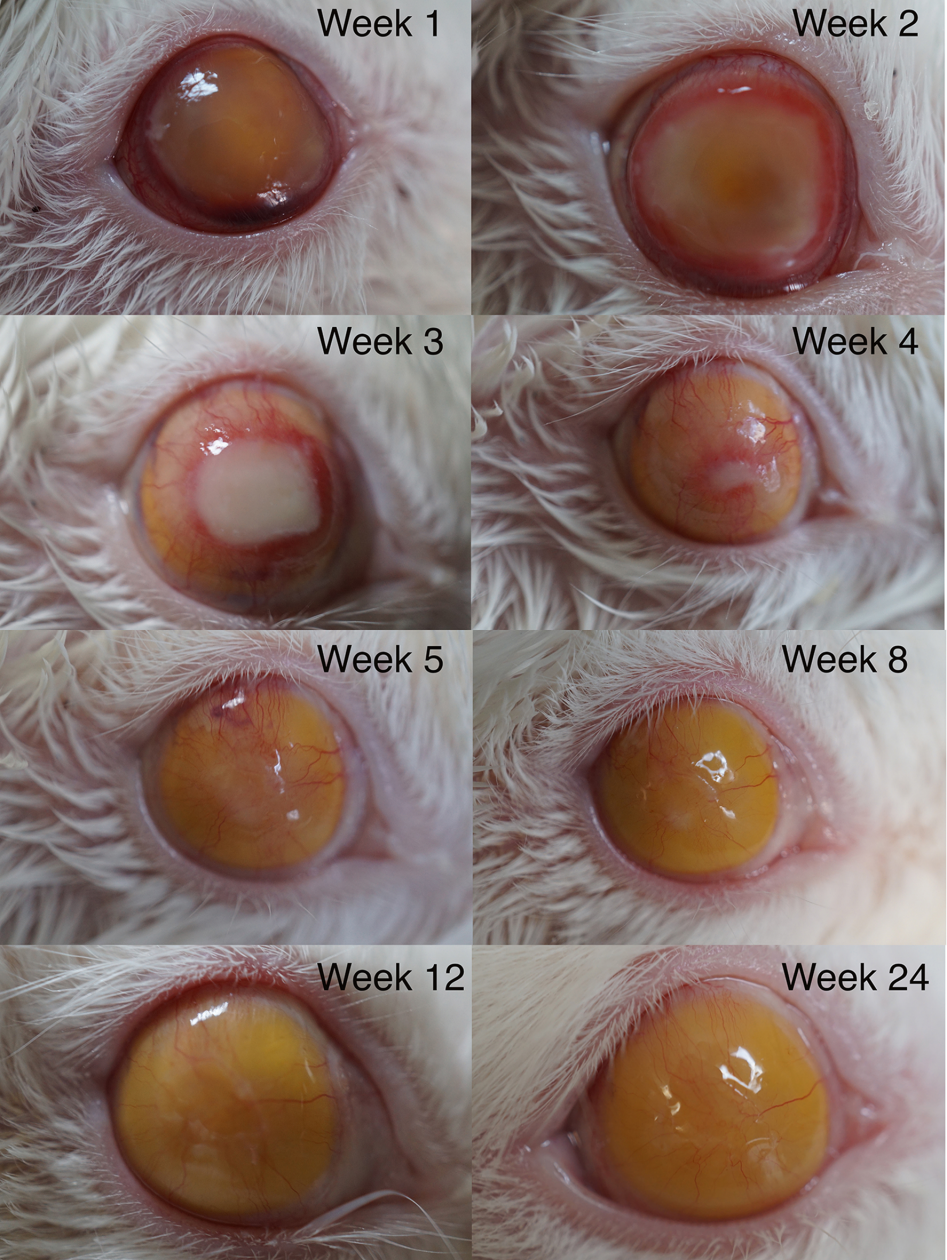
The photographs of the anterior segments. These anterior segment photographs showed the post-operative dynamic changes in the cornea. One week post-op, the cornea was transparent. No cornea epithelial edema or inflammatory reactions were noted. At post-op Week-2, the ring-shaped new vessels with significant cornea epithelia edema were observed in the peripheral cornea, which persisted for two weeks and invaded into the center cornea by Week-3. At post-op Week-4, the corneal pannus and edema regressed and the corneal transparency resumed. The injected ATLSRs were clearly visible through the cornea. From Week-5 to Week-24, corneal neovascularization regressed gradually and the cornea remained clear with intact epithelia.

The rabbits were assessed daily within two weeks post-operatively. Normal eye opening on both sides was noted in all rabbits. No signs were noted to suggest pain; nor abnormal scratch behavior was noticed. One week post-operatively, the corneas were transparent with intact epithelia apart from congested perilimbal vessels. The injected ATLSR was clearly visible through the cornea. At 2 weeks post-op, the corneas became edematous with significant limbus congestion, although the epithelia were intact. Neovascularization was observed in the peripheral area of the cornea. At 3 weeks post-op, neovascularization encroached into central corneal stroma with a ring-shaped network, and significant epithelial edema was noted. On the other hand, neovascularization in the peripheral cornea started to regress at this juncture with resolution of epithelial edema noted. At Week-4 post-op, pannus formation was noted in central cornea with some residual edema. The edema regressed mostly and the internal silicon rubber was visible through the cornea. Prominent vessels were found in the cornea stroma. At post-op Week-5, the cornea pannus and edema regressed completely. The stromal new vessels thinned markedly. At the Week-8 post-op, the corneal transparency resumed completely with intact overlying epithelia. From Week-12 to Week-24, the cornea remained clear with intact epithelia (Fig 1). The shapes of the experimental eyeballs were well formed as compared to the control eyes throughout the whole observation period up to Week-24, before enucleation was performed.

### 3.2 Diameters of the eyeballs

The diameters were measured for both the experimental and control eyes after enucleation (Table 1). Compared to the control eyes, the experimental eyes were significantly smaller in vertical diameters (17.00±1.17 vs. 17.54±1.11 mm, P<0.001), but larger in sagittal diameters (16.85±1.48 vs. 16.40±1.38 mm, P = 0.008), and had no significant differences in horizontal diameters (17.49±1.53 vs. 17.64±1.21 mm, P = 0.34) (Table 2).

**Table 1:** The diameters of the experimental and the control eyes.

No.	A1	A2	A1/A2	B1	B2	B1/B2	C1	C2	C1/C2
1	17.10	17.41	0.9822	18.30	17.52	1.0445	17.13	17.08	1.0029
2	17.04	17.71	0.9622	17.89	18.62	0.9608	17.55	16.67	1.0528
3	17.37	18.36	0.9461	18.14	18.48	0.9816	17.58	17.66	0.9955
4	17.94	18.21	0.9852	17.91	18.43	0.9718	18.14	17.88	1.0145
5	17.66	17.75	0.9949	17.45	17.58	0.9926	16.53	16.67	0.9916
6	18.86	19.30	0.9772	19.53	19.09	1.0230	19.05	17.98	1.0595
7	18.15	18.76	0.9675	19.11	18.70	1.0219	18.49	17.19	1.0756
8	17.33	18.06	0.9596	18.98	18.55	1.0232	17.27	17.03	1.0141
9	16.12	16.28	0.9902	15.11	15.76	0.9588	14.66	14.06	1.0427
10	14.88	15.78	0.9430	15.41	15.78	0.9766	15.30	14.62	1.0465
11	15.32	16.00	0.9575	15.35	16.08	0.9546	14.44	14.56	0.9918
12	16.18	16.90	0.9574	16.64	17.08	0.9742	16.10	15.38	1.0468

A1: The vertical diameter of the experimental eyes. A2: The vertical diameter of the control eyes. B1: The horizontal diameter of the experimental eyes. B2: The horizontal diameter of the control eyes. C1: The sagittal diameter of the experimental eyes. C2: The sagittal diameter of the control eyes.

**Table 2:** The mean diameters of the experimental and control eyes.

Group	The vertical diameter(mean±SD, mm)	The sagittal diameter(mean±SD, mm)	The horizontal diameter(mean±SD, mm)
Experimental eyes (n = 12)	17.00±1.17	16.85±1.48	17.49±1.53
Control eyes (n = 12)	17.54±1.11	16.40±1.38	17.64±1.21
t value	-6.544	3.221	-1.007
P value	<0.001	0.008	0.335

### 3.3 Histopathological study

**Fig 2:**
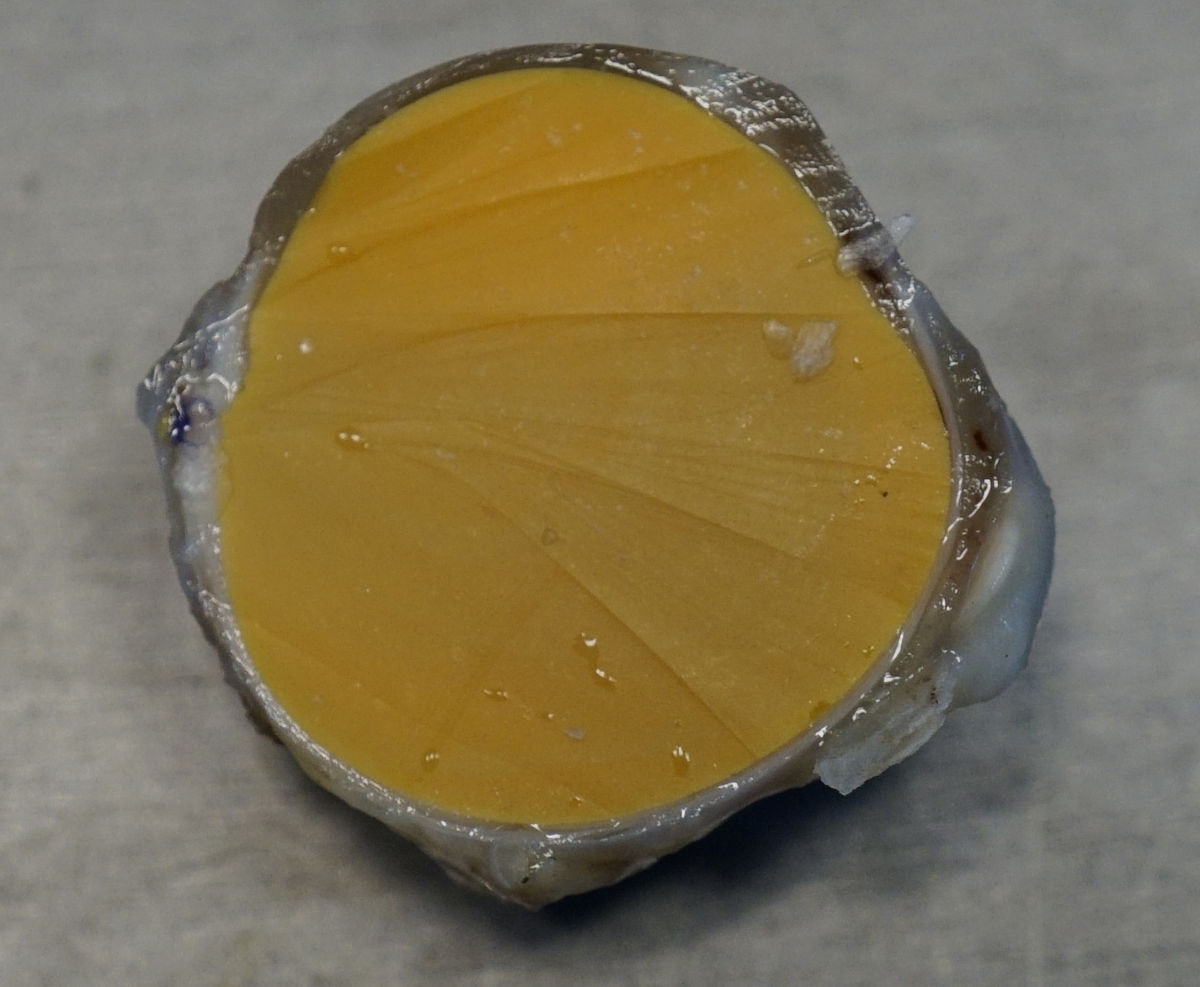
Cross-sectional image of an experimental eye. The experimental eye was completely filled with the solidified addition type liquid silicone rubber with preservation of the normal globe morphology.

**Fig 3:**
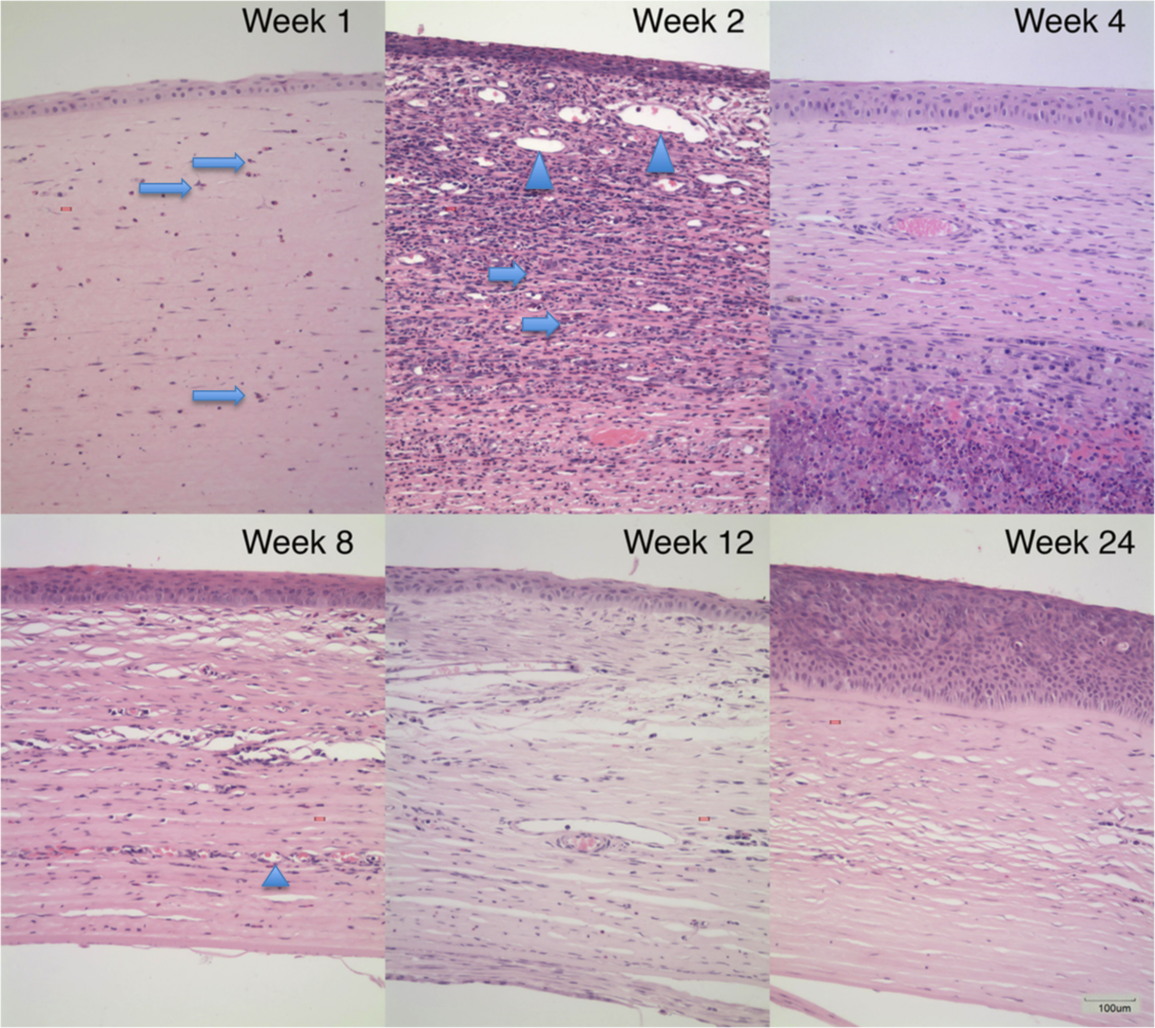
The histopathological photographs of the experimental eyes. The histopathological slides of cornea and sclera were stained with hematoxylin and eosin and were examined under light microscope. Main abnormalities included inflammation infiltration and neovascularization, which showed dynamic changes with time after surgery. Some neutrophils in the cornea and the sclera stroma were observed at Week-1 (arrows). At Week-2, neovascularization (arrowheads) and inflammatory cell infiltration (arrows) was observed in the cornea, which were mainly eosinophil and plasma cells. The inflammation cells persisted up to Week-4, with extensive new vessels observed. At Week-8, the inflammatory cells regressed markedly. Some vessels (arrowheads) remained in the cornea stroma. At Week-12 and Week-24, most of the inflammatory cells disappeared with only occasional plasma cells and eosinophils.

On gross examination, the experimental eyes appeared normal morphologically with intact cornea and sclera. The eyeballs were completely filled with ATLSR without any underfill (Fig 2). The histopathological study showed some neutrophils in the cornea and the sclera stroma at Week-1. At Week-2, neovascularization was seen in the cornea stroma. An abundance of inflammatory cells infiltration was observed in the cornea and sclera stroma, which were mainly eosinophil and plasma cells. The inflammatory cells persisted into Week-4, with extensive neovascularization observed. At Week-8, the number of eosinophil and plasma cells decreased markedly although some neovascularization persisted in the cornea stroma. At Week-12 and Week-24, most of the inflammatory cells disappeared with only occasional residual plasma cells and eosinophils. Some residual vessels persisted in the corneal stroma (Fig 3).

## 4.0 Discussion

In the current study, we used ATLSR rubber as the injectable implant. Compared to solid implants, either made of porous or non-porous material, the advantage of ATLSR is that it is available in an injectable liquefied state, which renders it easy to manipulate, and can fill the eyeball socket completely without any gaps. This ensures superior restoration of the morphology in the orbit [17]. Compared to other injectable liquefied materials such as silicone oil, ATLSR solidifies shortly after being injected, rending it easier to conform to the required shape. It is also more stable than remaining in the liquefied status [20–22]. The current study demonstrated that injection of ATLSR after evisceration could fill the eyeball completely and maintain eyeball volume and cosmetically satisfactory when compared to the fellow eye.

Yeatts et al first introduced a small incision evisceration with an injectable hydroxyapatite (HA) paste as an injectable ocular implant [17]. The preoperative mean axial length of the rabbit eyes in this study was 15.8±0.9 mm. The average axial lengths and lateral globe measurements of the operated eye were 3.1±0.7 mm less than the fellow eye 6 months after surgery (around 20%). Histological examination showed a decrease in granulomatous inflammation and an increase in vascularization of the implanted HA over the period with early osseous metaplasia. Koreen et al introduced 2.5% polyacrylamide hydrogel as an injectable ocular implant [23]. The relative diameters of the eviscerated eyes compared with control were 89 ± 6% (mean percentage ± standard deviation) at 12 weeks and 93 ± 4% at 1 year. The implant material was well tolerated with moderate giant cell reaction seen at 6 weeks, which improved over time. Extensive vascularization of the implant was noted beginning at Week-6. Possible resorption of components of the cement mixture and hydrogel, including the water diluent, sodium phosphate solution, or of HA, might have caused the shrinkage of the eviscerated globe.

Our study showed that ATLSR might be an alternative option as an ocular implant following evisceration. Post-operative clinical examination showed a cosmetically satisfactory appearance of the eyeball. Quantitatively, compared to the fellow control eyes, the experimental eyes were significantly smaller (3%) in vertical diameter, but larger in sagittal diameter, and had no significant difference in horizontal diameter. This minor deformation of the eyeball morph006Flogy was of no clinical implications. This might be caused by the removal of the traction from the internal membranes such as iris and ciliary body, or by the tension force of the injected silicone rubber, which deformed the eyeball from an ellipsoid shape into a spherical shape.

Postoperative inflammation was observed at one week after surgery in our study, which peaked at 2 to 3 weeks followed by regression gradually. It is hard to differentiate whether the inflammation was caused by the silicon rubber or the surgical procedure. Histologically, the inflammatory response is characterized by edema of the tissue with an infiltration of inflammatory cells such as neutrophils (in the short-time) or other lymphocytic cells (in the long-time) [24]. Since the infiltrated cells were mainly eosinophils and plasma cells, we speculated that the inflammation might be at least partially induced by the injected ATLSR. However, the inflammatory cells and the new vessels regressed markedly at week 24, suggesting potential long-term biocompatibility. Further studies are required to investigate the main component that incited the inflammation, such that the composition might be adjusted accordingly.

Addition silicon rubber (silicon impression) is widely applied in dentistry. It is not designed for ophthalmology. By changing the composition of the raw material, we can decrease the viscosity to facilitate faster injection via smaller incision. Setting time can be prolonged by lowering the temperature of the materials, thereby permitting adequate cavity filling.

In addition, extensive vascularization of the implant could induce opacities in the cornea. As the vascularization was limited, the cornea was preserved and remained transparent in this study. In real life, there is a possibility of the patient to wear a tinted contact lens for cosmetic purposes. In the future, if ATLSR implants of various colors are available, tinted contact lenses might not be even required as the color of the ATLSR implant could match that of the normal fellow eye.

There were a few limitations in this study. Firstly, the post-operative observation period is relatively short (i.e. up to 24 weeks). Long-term effects of the implantation need to be further determined in future studies with a longer follow up period. Secondly, the preoperative measurements were not performed for the experimental eyes. The comparison of eyeball morphologies before and after surgery was therefore not possible. However, previous studies have shown that eyeball shapes and sizes between the two eyes of any individual animal are usually not significantly different. And since the surgery is mainly for cosmetic purpose, similarities between the two eyes are perhaps more important than the pre and post-operative differences in a single eye.

### 4.1 Conclusion

In this animal model study, injectable addition type silicon rubber may be an alternative option for ocular implantation after evisceration, being able to maintain the eyeball volume and producing cosmetically satisfactory outcomes when compared to the fellow control eyes. Spontaneous regression of inflammatory reaction post-operatively suggested biocompatibility for at least 24 weeks.

## Supporting information

S1 File NC3Rs ARRIVE guidelines checklist 2014. (DOCX)

S2 File Ethical approval form. (PDF)
