# Supplementary material for: Injectable silicone rubber for ocular implantation after evisceration
Source: PLoS One. 2018 Mar 23;13(3):e0193448. doi: 10.1371/journal.pone.0193448 (PMC5866100; doi:10.1371/journal.pone.0193448)

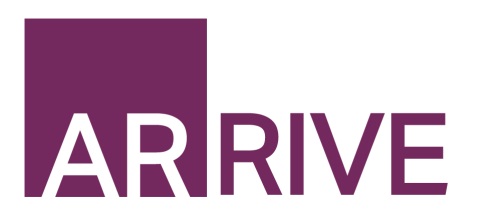


The ARRIVE Guidelines Checklist

Animal Research: Reporting In Vivo Experiments

Carol Kilkenny^1^, William J Browne^2^, Innes C Cuthill^3^, Michael Emerson^4^ and Douglas G Altman^5^

*^1^The National Centre for the Replacement, Refinement and Reduction of Animals in Research, London, UK, ^2^School of Veterinary Science, University of Bristol, Bristol, UK, ^3^School of Biological Sciences, University of Bristol, Bristol, UK, ^4^National Heart and Lung Institute, Imperial College London, UK, ^5^Centre for Statistics in Medicine, University of Oxford, Oxford, UK.*

|  | | ITEM | RECOMMENDATION | Section/ Paragraph |
| --- | --- | --- | --- | --- |
| 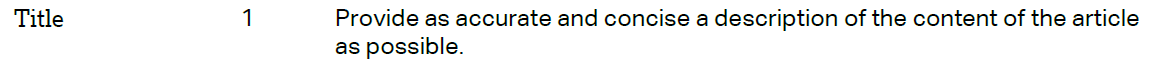 | | | Title |  |
| 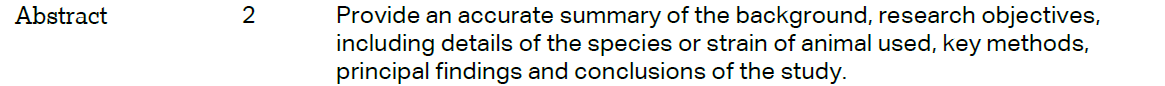 | | | abstract |  |
| INTRODUCTION | | |  |  |
| 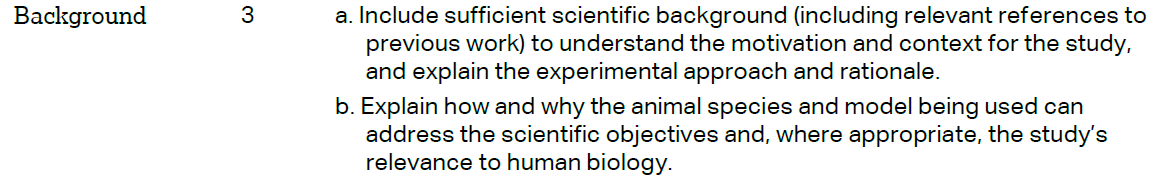 | | | introduction |  |
| 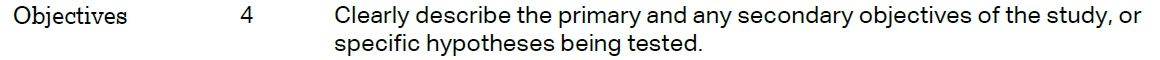 | | | Intraduction last sentence |  |
| METHODS | | |  |  |
| 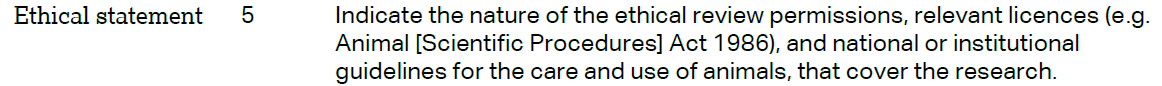 | | | 2.2 animal study, the first paragraph |  |
| 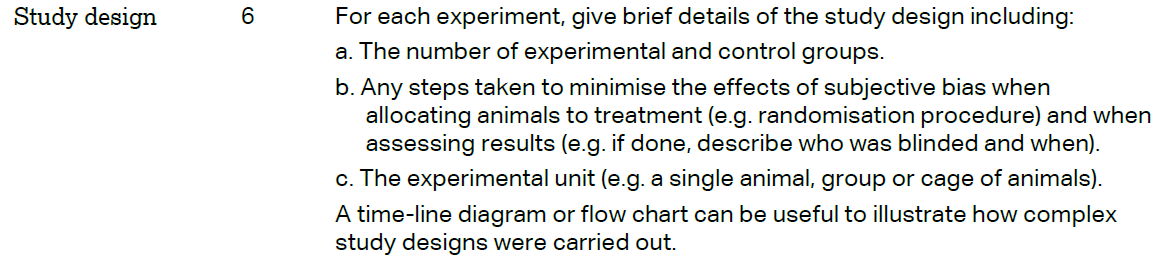 | | | 2.2 animal study, the second paragraph |  |
| 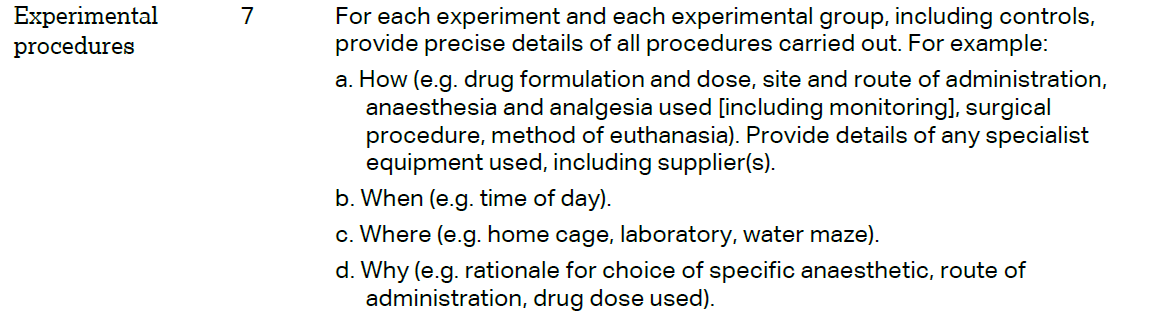 | | | 2.2 animal study, the second paragraph |  |
| 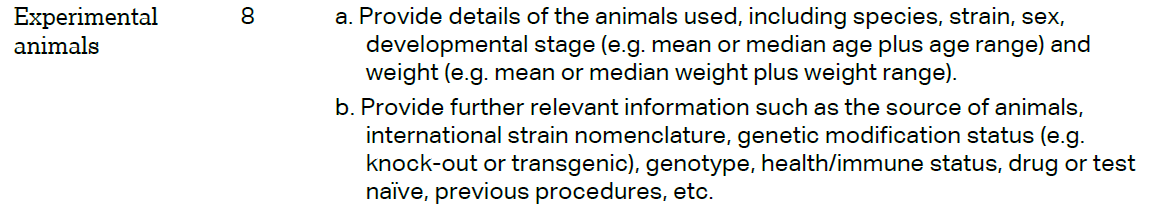 | | | 2.2 animal study, the second paragraph |  |

The ARRIVE guidelines. Originally published in *PLoS Biology*, June 2010^1^

| 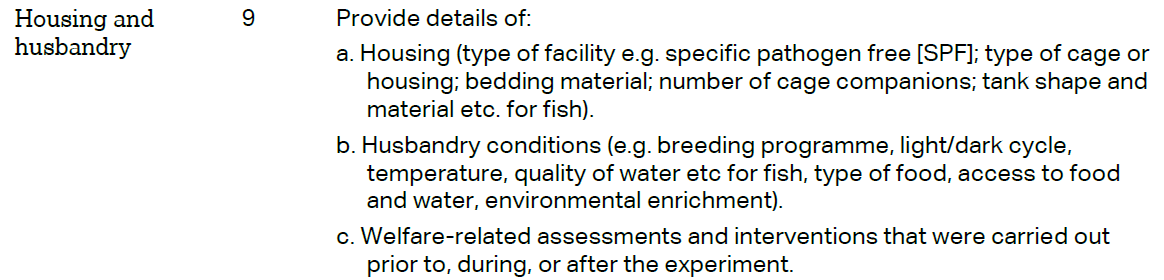 | 2.2 animal study, the second paragraph | |
| --- | --- | --- |
| 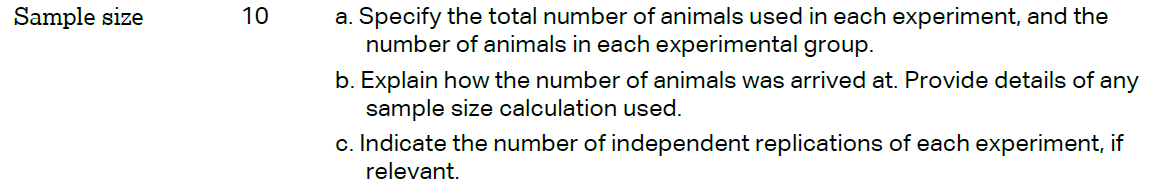 | 2.2 animal study, the second paragraph | |
| 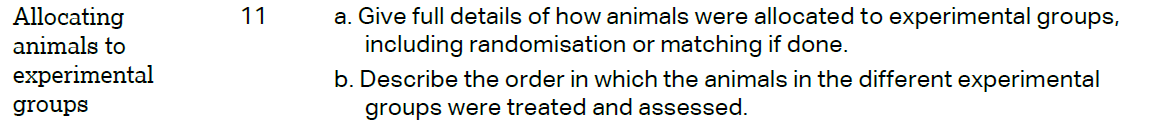 | 2.2 animal study, the second paragraph | |
| 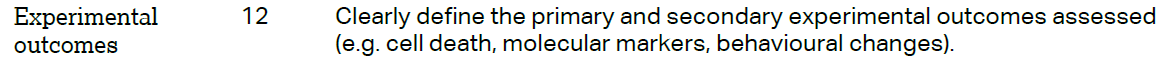 | 2.3Appearance and histological study | |
| 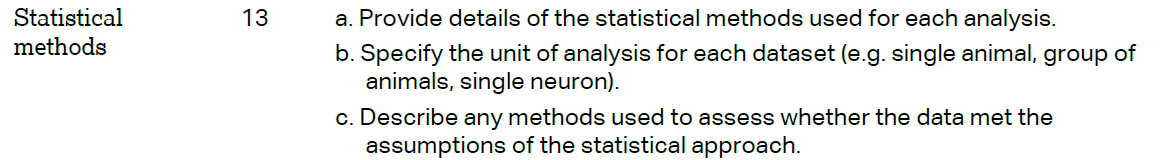 | 2.4 statistical analysis | |
| RESULTS |  | |
| 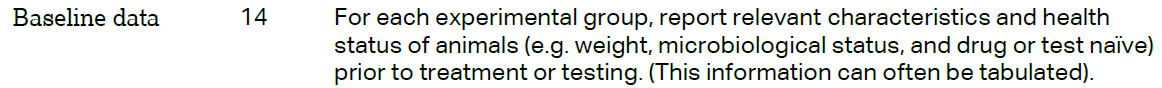 | Results 3.1 | |
| 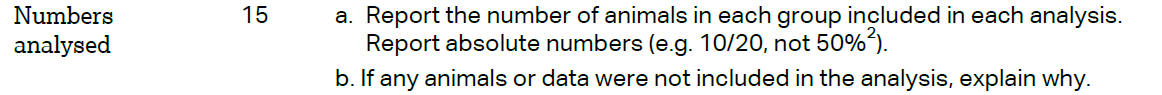 | Results 3.2 | |
| 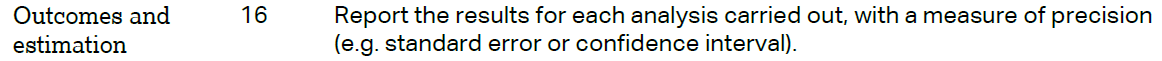 | Results 3.3 | |
| 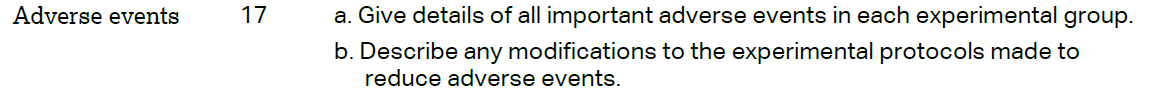 | Results 3.1 | |
| DISCUSSION |  | |
| 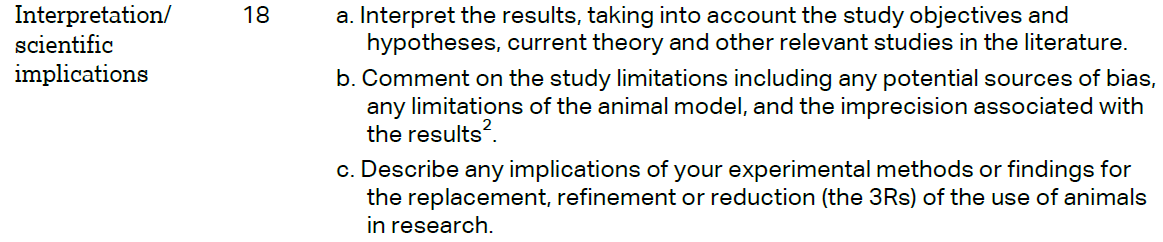 | Discussion  a-para 1,2,3,4,5,6  b-para 7 | |
| 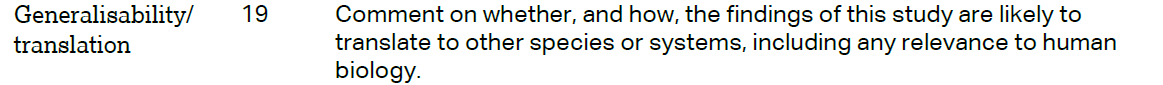 | Conclusion | |
| 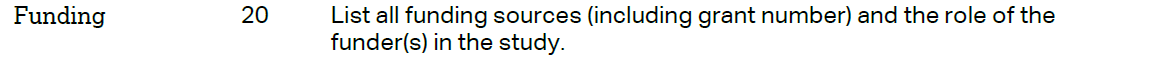 | | Funding |


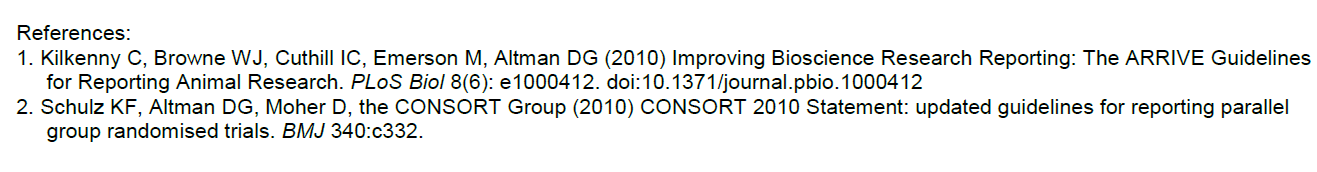

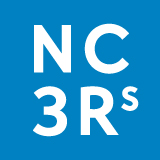

Supplement: S1 File — (DOCX) [file pone.0193448.s001.docx]
