# Supplementary material for: Injectable silicone rubber for ocular implantation after evisceration
Source: PLoS One. 2018 Mar 23;13(3):e0193448. doi: 10.1371/journal.pone.0193448 (PMC5866100; doi:10.1371/journal.pone.0193448)
Supplement: S2 File — (PDF) [file pone.0193448.s002.pdf]

首都医科大学实验动物使用申请表
(Application Form for Use of Laboratory Animals in Capital Medical University)

伦理编号:AEEI-2014-133

A 项目资料(Project Information)

1.	项目负责人姓名(Principal Investigator): 郑鹏飞	学院(College): 同仁医院
2.	学系(Department): 眼科	职称(Title): 副主任医师
3.	邮件地址(E-mail Address): trzpf@sina.com	办公电话(Office Phone): 58268256
4.	手机号码(Mobile Number): 13671031131	实验动物从业人员上岗证编号(ID): 201471600512
5.	项目名称及编号(Project name and Number): 眼内容剜除眼内充填物的研究	
6.	项目资金来源/卡号(Source of Funding/Card Number): 同仁医院科研基金	
7.	项目起止年月(Duration of Project): 2014.11.-2015.11.	
8.	项目总经费(Total project funding): 5万元	
9.	项目性质(Project nature): 校局级	
10.	项目子课题(Sub-project): 无	

B 项目子课题负责人 (Principal Investigator)

11.	子课题名称(Name of Sub-project):	
12.	姓名(Name of researcher):	
13.	学院(College):	学系(Department):
14.	职称(Title):	邮件地址(E-mail Address):
15.	办公电话(Office Phone):	手机号码(Mobile Number):
16.	实验动物从业人员上岗证编号(ID):	

C 实验人员信息 (Laboratory Personnel)

17.姓名 (Name of researcher)	实验动物从业上岗证编号(ID)	办公地点 (Office location)	项目分工 (Duty)	电子邮件 (E-mail)	电话 ((Phone)	备注 (Remark)
邓宏燕	2014071600132	北京口腔医院	动物实验	swallow.deng@163.com	13439342508	

D 动物使用情况(Animal Usage)

18.品种 (Breed)	品系 (Strain)	级别 (Grade)	年龄 (Age)	体重 (BodyWeight)	性别 (Sex)	预计总数量 (TotalNumber)
兔	新西兰兔	普通级		2KG	雌雄不限	12

E 动物饲养方式(Animal Housing)

19.饲养方式 普通饲养(Common) (√) 干养(Dry-feeding) ( ) 代谢笼(Metabolic Cages) ( ) IVC(Individual Ventilated Cages) ( )

20.是否需要单笼饲养? Will the animal be fed separately? (√) 是YES ( ) 否NO

如是，请说明 (If Yes, provide the following: please describe details and scientific justification below:) :
单笼饲养

F 动物转移 (Transportation Methods)

21.动物是否需要转到设施外实验室进行实验 (If animals need to go outside of the laboratory experiment facilities) ? (√) 是YES ( ) 否NO

如"是"，请描述理由(If "Yes", please describe reasons):
眼部辅助检查 (B超，核磁)

22.如果问题21回答"是"，动物是否继续饲养 (If you answered "yes" to 21, do live animals continue to feed) ? ( ) 是YES (√) 否NO

G 研究综述 (Literature Review)

23.简要介绍研究目的，阐述研究意义 (Please briefly summarize the aim of the study)

严重眼外伤、绝对期青光眼患者，患眼无视力且眼痛需摘除眼球。现有技术完整摘除，术后义眼运动差，外观欠佳。我们设计一种全新术式，行眼内容剜除并将可体内存留物充填于眼内以维持眼球外观。目前可用充填物仅有硅油一种，因为液态则维持外观稳定性差。因此我们设计将硅橡胶可固化物用于眼内。因此材料从未用于眼内，需实验动物研究

由 扫描全能王 扫描创建

H 实验动物福利信息（Animal Welfare Act Required Information）

24. 解释说明使用实验动物开展活体动物实验的必要性，是否可使用体外实验替代，如：数学模式、电脑模型、体外生物学系统等（Please explain the necessity for animal use, if it can be replaced by in vitro experiment, such as mathematical model）。

新术式、新材料用于眼内充填需实验动物了解其可行性及新材料对眼球壁组织病理学影响。本实验为前瞻性、描述性研究。充填物为硅橡胶，术后1、2、4、8、12、24周分批获取标本了解组织病理改变。

25. 解释说明使用该物种的合理性（Please justify appropriateness of each species selected and why phylogenetically lower or other species are not used）。

实验主要了解充填后眼球饱满度，如实验动物眼提及较小则手术难度大，误差大。兔眼体积适中，眼球壁组织学与人类无明显差异，且饲养易，价廉。

注：IACUC指的是实验动物使用和管理委员会（首都医科大学动物实验及实验动物管理委员会）。
IACUC, Institutional Animal Care and Use Committee.（Capital Medical University Animal Experiments and Experimental Animals Management Committee）

I 药品管理（Drug Administration）

26. 镇痛和麻醉药物Anesthesia and Analgesia,

品种<br>(Breed)	药品<br>(Drug)	剂量<br>(Dose)	途径<br>(Route)	频率<br>(Frequency)
兔	氯胺酮	30mg/kg（首次，20min后半量追加）	肌注/静脉	约1次/20min

27. 实验用药物（不包括麻醉剂，止痛剂和镇静剂）Drugs Administered for Experiment (excluding anesthetics, analgesics, and tranquilizers)

名称（中英文）<br>(Drug Name)	缩写<br>(Abbreviation)	剂量<br>(Dose)	途径<br>(Route)	药物使用过程中对其他人员或动物的危害(Harm to personnel and animals in the process of drug use)
硅橡胶(silicon impression)	SI	4ml	眼内充填	无害

J 安乐死（Euthanasia）

28. 实验结束后是否处死动物（Will the animals were killed after the end of the experiment?）？ （ ✓ ）是YES （ ）否NO

如果是，请选择安乐死操作方法(If Yes , Please provide the method of Euthanasia):

品种	品系	级别	年龄	体重	性别	安乐死
兔	新西兰兔	普通级		2KG	雌雄不限	过量麻醉处死法

如果否，请说明理由（If "No", please describe the reasons）？）：

K 实验设计及动物操作说明（Description of Experimental Design and Animal Procedures）

29. 提供一个流程图或表格，简明扼要的研究设计细节，特别说明实验中使用的动物总数和每个实验组中分配的动物数。（Please provide a flow chart or table , Clearly and conciselydescribe the animal used in the program, particularly the animal number of total and each experimental group used in the experiment.）

【文件链接】

30. 是否采血（Will blood sampling be conductedcollected）？ （ ）是YES （ ）否NO

如果是，提供理由，方法，采血量和频率（If Yes, Please provide rationale, method, site, volume, & frequency）：

31. 是否限制饮食或特殊饮食（标准手术前禁食除外）（Will food scheduling or restriction (other than standard pre-operative fasting) be conducted）？ （ ）是YES （ ）否NO

如果是，提供理由，方法，频率及持续时间（If Yes, Please provide rationale, method, frequency, & duration:）：

32. 是否限制饮水或特殊饮水（Will water scheduling or restriction be conducted ）？ （ ）是YES （ ）否NO

如果是，提供理由，方法，采血量和频率（If Yes, Please provide rationale, method, site, volume, & frequency）：

33. 是否长时间保定动物（Will restraint methods be utilized）？ （ ）是YES （ ）否NO
注：短时保定，如以采血为目的的简单保定不在此范围（Note: Restraint in this instance is defined as more than momentary. Brief restraint for the purposes of blood collection would not qualify as a restraint method）。

如果是，提供理由，方法，采血量和频率（If Yes, Please provide rationale, method, site, volume, & frequency）：

34. 是否有应激模式（Will stress paradigms be utilized）？ （ ）是YES （ ）否NO

如果是，提供的理由，类型，频率和持续时间（If Yes, Please provide rationale, type, frequency, &, duration）：

35. 手术是否存在致死性?（Will non-survival surgery be performed?） 注：IACUC认为麻醉后进行灌注或活体组织采集属于致死性手术（Please note that the IACUC classifies perfusion of or tissue collection from a live, anesthetized Animal as a non- （ ✓ ）是YES （ ）否NO

由 扫描全能王 扫描创建

survival surgery.）。	
如果是，请说明理由（ If "Yes", please describe the reasons）？	
36. 是否对同一动物进行多次手术（Will multiple survival surgeries be performed on one animal?）？	( )是YES (✓)否NO
如果是，请说明理由（If "Yes", please describe the reasons）？	
同一动物不能同时进行多次重大手术。如果需要，必须提供科学依据（If you are requesting to perform multiple major survival surgeries, Please provide substantial scientific justification below.）。	
37. 手术操作者姓名（Name(s) of Surgeon(s)） 郑鹏飞	
手术操作者是否做过此类手术（Has the surgeon(s) performed this procedure on the species requested）？	(✓)是YES ( )否NO
如是，请说明相关经历(If yes, please describe the relevant experience)	
38. 术后的护理和观察（评估/减轻疼痛/压力的方法，监测标准）（Postoperative care and observation (Methods to assess/alleviate pain/distress, recovery criteria, monitoring criteria.）。<br>理论上如无感染不致术后疼痛，行动异常可给予镇痛剂	
39. 细胞/肿瘤系/体液是否会在活体动物体上使用？Will cells, tumor lines, or bodily fluids be introduced to the live and/or dead animals?	( )是YES (✓)否NO
1、请说明如何确定仁慈终点?Please explain how to determine the humane endpoint?	
2、如果是，请提供以下信息：（If Yes, Please describe the cells/tumor lines/bodily fluids in the table below）	

种类<br>(Species)	试剂<br>(Agents)	剂量<br>(Dose)	途径<br>(Route)	作用<br>(Effect)

40. 是否要通过摄影，录像或其他记录设备获取图像？（Do you plan to capture any animal images via photography, video or other recording devices as part of your research project?）	( )是YES (✓)否NO
请说明获取图像的理由、方法、频率（Please explain the reasons for acquiring an image, the method, frequency）：	

声明：我将自觉遵守实验动物福利伦理原则，随时接受委员会的监督和检查，如违反规定，参照《首都医科大学动物实验及实验动物管理规定》自愿接受处罚。

（Statement: I will abide by ethical principles of animal welfare, ready to accept the Committee's oversight and inspection, such as the violation of the regulations, refer to animal experiments and experimental animal administrative regulations of Capital Medical University, voluntary acceptance of punishment.）

项目负责人签字：郑鹏飞

2014/12/21

首都医科大学动物实验及实验动物福利委员会意见：

（Comments of Animal Experiments and Experimental Animal Welfare Committee of Capital Medical University）

签字（盖章） [illegible]

2014/12/24

由 扫描全能王 扫描创建
